# Cultural adaptation of clinic-based pediatric hiv status disclosure intervention with task shifting in Eastern Uganda

**DOI:** 10.1186/s12981-025-00743-7

**Published:** 2025-04-19

**Authors:** Joseph Kirabira, Janet Nakigudde, Keng-Yen Huang, Scholastic Ashaba, Harriet Nambuya, Yesim Tozan, Lawrence H. Yang

**Affiliations:** 1 Department of Psychiatry, Faculty of Health Sciences, Busitema University, Mbale, Uganda; 2 Department of Psychiatry, Makerere College of Health Sciences, Kampala, Uganda; 3 Department of Child and Adolescent Psychiatry, Grossman School of Medicine, New York University, New York, USA; 4 Department of Psychiatry, Mbarara University of Science and Technology, Mbarara, Uganda; 5 Department of Pediatrics, Jinja Regional Referral Hospital, Jinja, Uganda; 6 Department of Global and Environmental Health, School of Global Public Health, New York University, New York, USA; 7 Department of Social and Behavioral Sciences, School of Global Public Health, New York University, New York, USA

**Keywords:** Adaptation, HIV disclosure, Caregiver, Feasibility, Acceptability, Task-shifting

## Abstract

**Background:**

HIV status disclosure remains a major challenge among children living with perinatally acquired HIV with many taking treatment up to adolescence without knowing their serostatus. This non-disclosure is influenced by factors like fear of the negative consequences of disclosure. Since HIV status disclosure has been found to have good effects including improving treatment adherence and better mental health outcomes, there is a need to design interventions aimed at improving disclosure rates among children living with HIV. This study aims at adapting a clinic-based pediatric HIV status disclosure intervention and tasking shifting from healthcare workers to caregiver peer supporters in Eastern Uganda.

**Methods:**

The adaptation process involved consultations with caregivers, healthcare workers involved in the care of children living with HIV, researchers in this field, intervention developers, and other experts and stakeholders. This was done through conducting FGDs with HCWs, caregivers, and peer supporters and consultations with researchers in the field of HIV. The original intervention manual was translated to Lusoga which is the commonly spoken dialect in this region. Collected qualitative data were analyzed using an inductive approach to develop themes and subthemes. Written informed consent will be obtained from all participants before participation in the study.

**Results:**

A total of 28 participants were involved in the FGDs, while two pediatricians and two HIV researchers/specialists were consulted. Six themes were generated in relation to all suggested changes to the original manual which were related to: (1) sociocultural beliefs/norms/perceptions (5 FGDs), (2) boosting caregiver’s confidence for disclosure (5FGDs), (3) disclosure mode, environment, and person (4 FGDs), (4) health facility/system related changes (3 FGDs), (5) reorganization/paraphrasing (3FGDs) and (6) age appropriateness (2FGDs).

**Conclusion:**

This study emphasized that whereas some aspects of intervention can apply to various contexts, there is a need for cross-cultural adaptation of interventions before being implemented in settings where they were not developed.

**Supplementary Information:**

The online version contains supplementary material available at 10.1186/s12981-025-00743-7.

## Background

HIV among children is mainly vertically transmitted during pregnancy, childbirth, or breastfeeding [1, 2]. By 2022, children up to 18 years of age accounted for about 6.6% of the global HIV burden, with a daily infection rate of 740 children [3]. By 2021, Uganda had 88,000 children aged < 14 years living with HIV and about 4000 AIDS-related deaths annually [4]. In this era of anti-retroviral therapy, many of these children survive into adolescence and young adulthood [5]. This comes with several health policy and service-related challenges regarding HIV testing and counseling services for this population, and several longstanding gaps persist in relation to disclosure and other areas [6]. In the early years of life, many children living with HIV (CLHIV) receive care without knowing their HIV status. This is often due to factors, such as stigma, fear of disclosing the mother’s HIV status, and the child’s young age [7, 8]. In Uganda, the recommended age for HIV status disclosure is 12 years [9]. The 2011 World Health Organization guidelines recommend that HIV status disclosure to children should start at age 8, with the process continuing incrementally based on the child’s cognitive abilities [10]. Nonetheless, all CLHIV should know their status by the age of 12. However, data from different settings show that HIV status disclosure can start as early as 6 years old or as late as 17 years old [11, 12].

Studies conducted in different low- and middle-income countries indicate that the prevalence of HIV status disclosure ranges from 1.7 to 41%, with nearly 50% of children receiving wrong information regarding their illness [13, 14]. In eastern and southwestern Uganda, this prevalence ranges from 31 to 56%, with non-disclosure and misinformation affecting up to 49% and 25% of children, respectively [12, 15]. Factors associated with non-disclosure include the fear that the child will disclose his/her status to others, the increased risk of stigma and discrimination, heightened feelings of hopelessness in the child, and concerns about negative psychological reactions, such as the child blaming the parents [13, 16]. Other factors associated with HIV status non-disclosure include lack of social support, the child’s young age (< 10 years), and parental reluctance to disclose to their partners, especially among women [14, 15, 17, 18]. Other barriers to HIV status disclosure among CLHIV include the absence of clear guidelines on disclosure interventions and a lack of knowledge by healthcare workers and caregivers on how to effectively disclose the child’s status [12, 18]. 

Despite the low prevalence of HIV status disclosure among children, research has shown that disclosure is more beneficial than harmful; it has been linked to improved adherence to HIV/AIDS treatment, better viral suppression, improved mental well-being of the children, and increased social support from teachers, family members, and other community members [14, 19]. Additionally, successful HIV status disclosure is a modifiable factor that can significantly improve children’s and adolescents’ engagement in HIV care [20]. This highlights the need for the World Health Organization (WHO) guidelines to incorporate HIV disclosure to children as a minimum standard in pediatric HIV care packages with relevant context-specific adaptations [21]. 

The current WHO guidelines, also adopted by the Ugandan Ministry of Health, recommend that pediatric HIV status disclosure should be carried out by the most trusted caregiver, with facilitation from a healthcare worker [22]. However, Uganda, like many other low-and middle-income countries, lacks context-specific disclosure interventions based on these guidelines and faces a shortage of healthcare workers due to limited health financing [6, 23]. As a result, healthcare worker-facilitated disclosure becomes unfeasible, as many people do not have access to these professionals. In Ghana, a clinic-based pediatric HIV status disclosure intervention was developed and proven effective in improving HIV status disclosure and mental health outcomes, such as depression among CLHIV and their caregivers [24]. The intervention was designed to be delivered in a personalized and age-appropriate manner by a well-trained clinician who is knowledgeable about the sociocultural norms of the community. However, in resource-limited settings like Uganda where there is inadequate staffing in the health sector, it becomes crucial to leverage other available resources and personnel to ensure timely service delivery. This makes the utilization of trained laypersons or volunteers—non-clinicians with good knowledge of local sociocultural norms—an ideal solution for delivering interventions. Such an approach help mitigate costs and reduces the work overload on the limited clinical staff. Therefore, this study aims to adapt the clinic-based pediatric HIV status disclosure intervention for implementation in HIV clinics in eastern Uganda, with shifting tasks from healthcare workers (clinicians) to caregiver peer supporters (CPS).

A CPS is a volunteer caregiver of a child or adolescent living with HIV who has successfully completed specialized training. This trained individual provides support or guidance to other caregivers as they go through the pediatric HIV disclosure process within the same HIV clinic. CPSs will be selected purposively based on their education level, regular clinic attendance, and good social and communication skills, as assessed by healthcare providers at the HIV clinics. Additionally, selected caregivers must have personally completed the HIV disclosure process with their children or adolescents, allowing them to draw on their own experiences to support others. By utilizing CPSs, we can address the challenges arising from inadequate health funding, staffing shortages, and the workload burden on clinical staff, while also promoting HIV disclosure among children and adolescents living with HIV. We qualitatively examined the stakeholder and other contextual factors that may influence the adaptation and implementation process of CPSs utilizing a pediatric HIV status disclosure intervention within the Ugandan HIV pediatric care setting. Hence, the study aimed at adapting the clinic-based pediatric disclosure intervention (manual) developed in Ghana with task shifting from HCWs to CPSs in Eastern Uganda, based on the above factors.

## Methods

### Study design and setting

Qualitative methods were employed, including in-depth interviews and focus group discussions conducted among healthcare workers, caregivers of CLHIV, and other experts involved in HIV care among children. The Consolidated Framework for Implementation Research (CFIR) was used to identify stakeholders and factors that may influence the adaptation and implementation process of the intervention. This implementation research framework helps design implementation strategies for evidence-based interventions by identifying barriers and facilitators to implementation across multiple levels, thus guiding necessary adaptations. The CFIR categorizes relevant individual and systemic factors under five domains: intervention characteristics, outer setting, inner setting, characteristics of individuals (stakeholders), and the process of implementation [25–28]. 

The study was conducted at the pediatric HIV clinic of Jinja Regional Referral Hospital (JRRH) in Eastern Uganda. JRRH is a tertiary hospital providing both general and specialized surgical and medical services in outpatient and inpatient settings to people in the eastern region. The hospital provides HIV care to children through its HIV clinic in the pediatric department, primarily on an outpatient basis. They also work in collaboration with other service providers, such as government agencies, Uganda Cares, and the AIDS Supporters’ Organization.

### Study population, sampling, and recruitment

The study mainly involved conducting focus group discussions (FGDs) among all healthcare workers (HCWs) directly involved in the care of CLHIV at JRRH, including medical officers, clinical officers, midwives, and medical social workers. Other participants included young people and adolescent peer supporters (YAPS) and linkage facilitators who support HIV service delivery at JRRH. The YAPS and linkage facilitators are voluntary members of the community who support HCWs in the provision of services to their peers. They play a bridging role between health workers and community members. The YAPS are adolescents living with HIV and mainly support fellow children and adolescents living with HIV, while linkage facilitators are health community members who are selected to support adults living with HIV or caregivers of CLHIV in accessing services at the health facilities [29, 30]. 

Three separate FGDs were conducted among HCWs, YAPS, and linkage facilitators, and each FDG included 4 participants as these comprised all available persons for each category at the clinic. Additionally, two FGDs were conducted among caregivers of CLHIV attending the hospital’s pediatric department, and each FDG consisted of 8 participants. The participants were selected purposively with the help of HCWs at the clinic based on their regular clinic attendance, level of education, and ability to understand and discuss HIV-related concepts. Caregivers of CLHIV aged 8–16 years, who have been attending the participating HIV clinics for at least 6 months, were selected because they are more likely to be familiar with HIV care in the local context. Participants were recruited consecutively during routine clinic visits for their children’s care or were contacted by phone by the research assistants. A convenient day for each group discussion was arranged by the research assistants in coordination with the caregivers. Transport refunds and modest compensation for their time were provided to all participants.

### Adaptation process and data collection

The adaptation process followed the steps outlined in the systematic review by Escoffery et al., as described below [31]. We conducted a community assessment by engaging a broad range of stakeholders, including CLHIV and their caregivers, healthcare providers and service users at JRRH, health policymakers, administrators, and key community leaders. This was meant to ascertain the situation of HIV disclosure for CLHIV and assess the need for an HIV disclosure intervention. A literature search was then conducted about the existing HIV status disclosure interventions, and the clinic-based pediatric HIV status disclosure intervention was selected as the most appropriate evidence-based intervention for adaptation to our setting. This intervention was chosen because it was assessed as appropriate for the target population, was developed in an African setting, and focused on context-specific barriers to disclosure in all stages of the process (pre-disclosure, disclosure, and post-disclosure stages). Briefly, the core elements of this intervention are based on the information, motivation, and behavior skills model [32]. This model postulates that an individual’s ability to perform a health-related behavior depends on their information about the behavior (whether accurate or not), their personal attitudes and beliefs (motivation), and the skills—objective or perceived—required to perform that behavior [32, 33]. In this pediatric HIV disclosure intervention, caregivers are provided with accurate information about HIV status disclosure, addressing known barriers and facilitators. This information motivates them based on the anticipated consequences of disclosure or nondisclosure. Finally, their behavioral skills, such as effective communication with the child and the activation of social support, are assessed and improved to facilitate the disclosure process. While this model has been applied in the development of several health interventions, it underscores the role of other determinants of behavior, such as social and physical opportunities [34, 35]. The intervention also acknowledges that disclosure is a process, not a one-time event, and involves pre-disclosure, disclosure, and post-disclosure sessions between the HCW and the caregiver, followed by the sessions with the child [36]. In this context, the adaptation was aimed at ensuring sociocultural appropriateness, while also empowering caregivers of CLHIV who have successfully disclosed to take over the facilitation of the disclosure process from HCWs.

The next steps in the adaptation process involved identifying the necessary changes to the original intervention. This was done through conducting focus group discussions with key stakeholders, including HCWs, peer supporters, voluntary community health workers, and caregivers of CLHIV. Participants were purposively selected based on their knowledge and experience regarding the HIV status disclosure process among CLHIV and the sociocultural context. FGDs among HCWs, YAPS, and linkage facilitators were conducted in English, while those with caregivers were held in Lusoga (local dialect) based on participants’ preferences. The FGDs were conducted using the original intervention manual and potential changes were suggested with reasons. All sessions were audiotaped and transcribed verbatim by two independent team members.

In addition, individual consultative interviews were conducted with experts, including HIV researchers specializing in disclosure, a children and adolescent psychiatrist, and pediatricians familiar with the local sociocultural context. During these interviews, we reviewed the original intervention manual alongside the suggested changes from the FGDs. Based on the feedback from these consultations, decisions were made regarding which sections of the intervention required modification to better align with the needs and the context. These changes were primarily centered around cultural appropriateness and the feasibility of delivering the intervention by CPSs instead of HCWs. The review was led by a team of experts under the guidance of the principal investigator.

### Data management and analysis

Data from the FGDs was analyzed by thematic analysis following an inductive approach using N-Vivo software. The English audio recordings were transcribed verbatim by NEA while those in Lusoga were first transcribed verbatim by PW and then translated into English by RN. All transcripts were read several times by the researchers (JK and PN) to familiarize themselves with the data. The researchers then critically appraised the data and reflected on what it meant. Codes were generated for the segments that appeared meaningful about the study objective. Codes were then reexamined by the above two researchers independently to derive final codes by consensus or involving a senior researcher (SA). and potential subthemes were derived and later merged into major themes. The team kept on refining the themes during the analysis.

### Quality control and ethical considerations

Data was collected by well-trained research assistants rather than attending clinicians to minimize conflict of interest and allow participants to freely express their views, especially during the qualitative interviews. The research assistants who shared the same sociocultural background as the participants, were trained in administering all the data collection tools and conducting FGDs. All study tools and the intervention manual were translated into Lusoga to ensure consistency during data collection. The principal investigator regularly attended the discussion sessions to monitor the performance of the research assistants.

Ethical approval for the study was obtained from the Mbale Regional Referral Hospital Research and Ethics Committee and the Uganda National Council for Science and Technology. Additional administrative clearance for the study was granted by the JRRH administration. Participation was voluntary, and written informed consent was obtained from all study participants prior to their participation. All interviews and discussions were conducted in safe and secure locations to ensure privacy, and the data collected was handled with the utmost care to ensure confidentiality.

## Results

The study involved a total of five FDGs with 28 participants, with the majority being caregivers of CLHIV, while the consultations/interviews involved only 4 participants. (Table 1).

**Table:** **Table 1**

Consultations	Participants	Number of participants (*n*)
**Focus Group Discussions (FDG)**		
- FDG #1	Medical officer	1
	Medical clinical officer	1
	Medical social worker	1
	Midwife	1
- FDG #2	Young people and adolescent peer supporters	4
- FDG #3	Linkage facilitators (LF)	4
- FDG #4	Caregivers of CLHIV	8
- FDG #5	Caregivers of CLHIV	8
**Individual Interviews**		
	Pediatricians	2
	Child and adolescent HIV researcher	1
	Child and adolescent psychiatrist	1

### Themes and sub-themes of changes made to the intervention manual

Changes to the original intervention manual of the clinic-based pediatric disclosure intervention that were made fell under six themes, with several sub-themes identified in different FGDs. These six themes were: (1) Sociocultural beliefs/norms/perceptions (all FGDs), (2) Boosting caregiver’s confidence for disclosure (all FGDs), (3) Age appropriateness (only 2 FGDs), (4) Disclosure mode, environment, and person (4 FGDs), (5) Health facility/system related changes (3 FGDs), and (6) Reorganization/paraphrasing (3 FGDs). Each theme and sub-theme is described in detail below and Table 2 provides an overview of how they align with the CFIR domains. A summary comparing changes between the original and adapted intervention manuals is presented in Table 3 (Appendix 1) and a copy of the adapted intervention manual for CPSs is provided in the Supplementary Material for further reference.

**Table 2:** Overview of the alignment of different themes and sub themes with the CFIR framework

CFIR domain	Theme	Sub-theme
**Intervention characteristics**	Age appropriateness	HIV illness versus treatment Disclosure information
**Inner setting**	Sociocultural beliefs/norms/perceptions	Stigma and discrimination Social support systems Sex education
	Health facility/system changes	Identity Supervision/Referral Follow-up
**Outer setting**	Sociocultural beliefs/norms/perceptions	Stigma and discrimination Social support systems Sex education
**Characteristics of individuals**	Boosting caregiver’s Confidence for disclosure	Assessing caregivers’ HIV/disclosure-related knowledge and fears Assess disclosure readiness
**Process of implementation**	Disclosure mode, environment, and person.	
	Reorganization/paraphrasing	

### Sociocultural beliefs/norms/perceptions

The study participants suggested several changes to improve alignment with the social and cultural beliefs, norms, or perceptions of the local community about HIV and its treatment among children and adolescents. These changes were intended to minimize the rejection of the intervention by ensuring that the messages were culturally appropriate and well-perceived, ultimately increasing the uptake of the intervention. These changes were categorized into three subthemes.

#### Stigma and discrimination

Participants suggested changes that will minimize stigma and discrimination, demystify misconceptions, and ensure confidentiality of participant information. This is because stigma was also highlighted as one of the common reasons why caregivers may not want to disclose to children or even participate in this study.

*YAPS*: “*You mentioned cultural beliefs where someone has those negative beliefs where if you tell your child that you are this and this*,* he will think that he will die soon or think that people will start laughing at him or her.”*

*YAPS*: “*We have another girl where the neighbors know. From the place they started renting*,* they knew the child was HIV positive up to where they are now. And the girl has grown*,* she’s in secondary but they are still disclosing to others. So*,* she has reached a time when she does not wish to be home. She’s over discriminated against.”*

#### Social support systems

The participants suggested several changes to align the intervention with the local social support systems, ensuring that CPSs can communicate effectively to strengthen, rather than compromise, these networks. This includes parents and other relatives, regardless of their HIV status.

*HCW: “Still*,* it is those they live with. We are looking at the family support system. Whether the aunt*,* or grandparents. So*,* someone may tell you*,* no one in the family knows but my friends know. So*,* something wrong may be in that family if they can entrust the friend more than the family members.”*

*LF:” I think the option of “Is anyone else in the family having a similar illness?” may work. This works because according to my thinking because we want to support the child to know the reason for taking the drugs. He/she might say you see even this one is like this and he/she also takes the drugs; this will give him/her strength to keep taking the drugs because he/she will know why he/she is taking the drugs.”*

#### Sex education

Participants expressed concerns about the inclusion of sex education in the pre-disclosure and disclosure phases since it is a culturally sensitive issue. Many feared that this intervention may be misunderstood by caregivers as promoting sexual activity among young children.

*YAPS: For me even to ask my brother about his sexual life*,* I had a hard time. It is so uncomfortable. Sometimes it’s easy but your relative*,* eh. You become shy! So*,* our parents will not manage this one.*

*HCW: We do sex education in the clinic*,* but some parents fear it. They think you are giving go ahead to their children so it will be hard for them. They may think you only teach sex.*

However, some participants reported that they encountered sexually active children and adolescents in the clinics, though they still noted that it is difficult for caregivers to discuss sexual issues with children.

*LF:” There was a time we received a mother who had a child who was around 12 years old*,* and she was coming for family planning*,* and she knew exactly what kind she wanted so sex education is important and appropriate. The caregiver may choose not to tell but the child must have that information.”*

*LF:” Some child came and wanted condoms. So*,* she was like if you are not giving them to me*,* I will go live. So*,* I had to go look for condoms. Our children here are a bit open. They tell you*,* sister*,* I want condoms if you are not giving them to me*,* I will just have sex without it*,* so you are forced to give him.”*

After consultation, the consensus was that caregivers should decide whether to communicate practicing safe sex after determining whether their child is at risk of unsafe sex or not. Additionally, the information related to sex in the manual should be age-appropriate.

*YAPS: “For adolescents and children at risk for unsafe sex*,* tell them how to practice safe sex. You know the CPS is telling the parent*,* but not the child. He/she will leave it to the parent to tell the child. Do you think our parents can tell their children about sex? No! Some would but the majority*,* no. Like to use a condom*,* no.”*

*HCW: “Maybe we can put age-appropriate sex education. Because some are very young*,* but they’ve slept with more than 5 people. Here you need to bring it out. It depends on which kind of child you are dealing with. So*,* we could tell them so that they choose. But when we have equipped them with the information"*.

### Boosting caregiver’s confidence for disclosure

The participants suggested several changes to the original manual that aimed at boosting the caregivers’ confidence to participate in the study and the disclosure process. These were mainly related to assessing their HIV/disclosure-related knowledge, fears, and disclosure readiness.

#### Assessing caregivers’ HIV/disclosure-related knowledge and fears

This would involve understanding caretakers’ reasons for non-participation/non-disclosure, exploring their fears and concerns related to disclosure, and providing adequate information related to HIV/AIDS. Participants agreed that there is a need to seek clarification from participants who declined to participate in the study or disclose to their children. This is because the reasons may be addressable by providing adequate information, and these reasons may also influence other caregivers not to participate or disclose. Also, participants emphasized the need to explore caregivers’ fears during the pre-disclosure phase and provide adequate and accurate HIV/disclosure-related information to address them.

*HCW: “We need to know because sometimes things that bias them are things you can support them about and eventually*,* they agree to participate. It’s important to get clarification in what is stopping them from taking part as they may eventually influence the rest of the group.”*

*YAPS: “Me I think a parent doesn’t feel comfortable if he or she lacks information because ideally you can’t fail to explain to your child why she is positive yet now for her*,* she can give birth to a negative child. Maybe in the preparation you never gave the parent enough information or maybe the parent never asked for enough information. But if a parent is packed with enough information*,* he can’t fail to answer*,* whether it’s the middle child*,* first or last one. There must be an answer to why it is like that. Maybe when all of you don’t have but the child has*,* it can be through an accident.”*

The common reasons for non-disclosure highlighted by participants included poor communication, fear of negative emotions, fear of disclosure to others/child cannot keep secret, discrimination, and deception (for caregivers have previously lied to children about their illness).

*LF: “Most parents fear emotional damage*,* for example*,* one parent said that the child was once hurt*,* and he wanted to fall in water so what will happen if he knows this. What will he do? I got one after disclosing she cried so much after she told the uncle ‘Let me go for a short call’. After some time*,* I was like Uncle where has the child gone then the uncle found her seated crying. She never saw the mum or dad she had grown up with the grandmother and uncle.”*

*CAREGIVER1: “The only different idea I have is that for us parents it’s hard because we fear telling the children because the kids can easily tell other people. If I tell my child*,* will they keep the secret because children are not the same? What I think has made me fail to tell my child is the thinking that if I tell him the neighbors will know and it will affect him as am not always around. So that is the challenge*,* and I don’t know how we shall handle it”.*

#### Assess disclosure readiness

Participants noted that there was a need to incorporate a tool to assess both the child’s and the caregiver’s disclosure readiness in the pre-disclosure phase. This was believed to make it easy for all CPSs to determine who is ready or not before proceeding to the disclosure phase according to the existing Ministry of Health and WHO guidelines.

*HCW: And some will continue telling you that my child is not ready even up to 16 years. So*,* if we had a set of questions that would determine the readiness of this child for disclosure. I think that would help.*

*HCW: Maybe we should incorporate that disclosure readiness tool because it may be hard for the peer supporter to know if the child is ready.*

### Age appropriateness

Participants suggested several changes to the manual to ensure that the information was appropriate for children of different age groups. These modifications primarily focused on the pre-and-disclosure phases and were aligned with the following considerations.

#### HIV illness versus treatment

Participants highlighted that young children are more likely to ask why they are taking medicine as opposed to what illness they are suffering from.

*HCW: When we go back to has the child ever asked you about his/her HIV status*,* do they ask about the illness or the medicine? They ask about the medicine first. Well*,* sometimes it depends on whether they can ask why is it only me taking these drugs. What am I suffering from? They ask both. The medicine and why are they taking those drugs?*

*HCW: Because for those that are very young*,* usually they ask*,* why am I taking this medicine? But those that are above 12 years*,* want to know the kind of illness. We must be specific about the age group*.

#### Disclosure information 

Also, participants urged that the HIV status disclosure information should be both age-appropriate and socio-culturally acceptable. The information delivered should be easily understood by children.

*HCW: We can see that when it gets to the real process of disclosure. My assumption is we shall have different information and different age groups or ages so maybe we can see how best we integrate that when we get to step 4.*

*YAPS: I think for HIV transmission 13*,* 14*,* even 12 years at the least. The reason why 9 may be difficult is because most times by 9*,* a child will not understand clearly what you are talking about. I was 12 years but still*,* I was not sure what they were talking about. But sometimes they tell them when they are 12 or 13 when they at least got more information about it from the school. Even 12 but there are still some 12-year-olds who will not get it.*

### Disclosure mode, environment, and person.

Several suggested changes were related to the mode of delivery for disclosure information. Participants highlighted that the mode of delivery should be appropriate for different ages. They recommended using pictures and tables, role-plays, and toys to communicate information to the child.

*HCW: The same way we do for viral load. You know when you are talking to young kids*,* they have not been disclosed to*,* but you have these pictures. You see this young boy; how many viruses are in his body and then they will be able to tell the difference between these two pictures. And understand the situation.*

*HCW: So*,* there are things here we need to bring out to help them understand and so if it’s a role play*,* the session here we are now imagining the child before this caregiver and how this caregiver is going to approach or give up the information to the child. Some things cannot be left out. And they are the things that are going to make a difference.*

Additionally, whereas disclosure may take place in hospital settings, participants highlighted that other environments, such as the home, community, and school settings, could serve as favorable environments for disclosure depending on the child and caregiver’s preference. Caregivers alone may disclose to the child or request the presence of a CPS/HCW.

*YAPS: First*,* you find a convenient place*,* and keep a distance from the caregiver/guardian. Or if you are under the tree*,* you ask the caregiver for space and discuss with the child alone. And we discuss our things. We talk about other things.*

*CAREGIVER 2: For my case*,* I love to disclose in the school’s staff quarters but sometimes the matron shouts at the students in an open ground in the presence of other students which is not okay. Which causes stigma among students. Awareness of the school staff about stigma and HIV care in schools is very needed.*

*CAREGIVER 2: Disclosure should be in the presence of a health worker; it doesn’t matter where the place is.*

### Health facility/system changes

Participants suggested additional changes to ensure that the intervention aligns with the existing healthcare system and HIV care programs at the hospital. The aim was to ensure that the intervention runs in unison with the existing hospital programs and is seamlessly integrated into the hospital system.

#### Identity

Participants suggested that the intervention should be integrated into hospital activities so that CPSs identify with the existing hospital team.

*YAPS: Is the project going to be part of the hospital? Because if it is going to be part of the hospital automatically*,* they will be calling you a “Musawo” [local term meaning “healthcare worker”]. Where is that Musawo coming from? So*,* you will be mentioning*,* that I’m from Jinja Hospital*,* I am here to help you with your child’s sickness and help you with any other problem. For the parents*,* you don’t say that I’m from X*,* or that I’m from Y*,* some parents will hear and say*,* Y what is that*,* if they say Y*,* they will say eh*,* we are scared you will bring your cars here. You just say where you are from.*

*HCW: I also think that this introduction is not introducing the caregiver peer supporter. Yes*,* you have come but as who? Maybe if the person said my name is this and I am or I work as CPS in the hospital. Because we are replacing ADDS with what CPS. So*,* I work as a CPS at Jinja Regional Referral Hospital.*

#### Supervision/referral

Participants suggested that CPSs need to be attached to a HCW or hospital staff for supervision. This ensures they are monitored in terms of their progress, such as the number of caregivers they are supporting, and allows for additional support when needed. Additionally, all CPSs should be encouraged to refer caregivers whose conditions are beyond their capabilities. After consultation, it was agreed that the main referral/supervising person for all CPSs will be the social worker. However, HCWs would handle specific referrals based on the nature of the issue. Common reasons for referral included non-progressing caregivers, need for additional support, complications related to the child or caregiver, such as mental illness.

*HCW: Because my worry is. If we do not try to evaluate these peer supporters by maybe the number of caregivers supported*,* some will continue supporting them for much longer. But if you tell them at least the duration of the study*,* try to get some feedback from this caregiver*,* what is that which they’ve been able to do? Where do they need more support? It’s more like a follow-up. I don’t know if you have that session. But it’s more of a follow-up they have on some of the things they will have agreed with the caregiver.*

*YAPS*: *I think they should refer. Which is not a forceful referral. Because the parent has shown attitude*,* I think such a parent cannot show attitude without anything he or she is going through. I think the best thing is to refer to the parent they’ve for support or to a counselor within the facility.*

#### Follow-up

Participants emphasized that follow-up is an important component of the HIV disclosure process, especially during the pre- and post-disclosure phases. They recommended that CPSs should regularly follow up with caregivers and provide support, either via phone calls or in-person meetings.

In the pre-disclosure phase, follow-up was seen as key for monitoring caregivers as they make the decision to disclose. It provides an opportunity to offer additional information, address any concerns, and ensure caregivers feel supported and confident in their decision-making process.

*YAPS: they (CPS) follow up*,* they let her/him think about it*,* sleep over it*,* and see. So*,* they give her a call*,* asking how far*,* like if she continuously goes on refusing it’s her/his choice because they only advise. So*,* I think they give them some time to make a choice.*

Additionally, participants emphasized that post-disclosure follow-up should aim to address any psychosocial challenges that may arise from disclosure. This follow-up will be done immediately after disclosure; again after 2 weeks, and then on a monthly basis for 6 months, aligning with the existing peer support follow-up programs. Additionally, participants suggested that CPSs should assess the child’s adherence to treatment during these follow-up sessions.

*HCW: It is 2 weeks*,* 4 weeks*,* so we can do two weeks and 4 weeks for the next 6 months. Usually*,* the first month has so many issues but for those that we have disclosed to*,* the practice here has been we attach a YAPS and there’s a month with support. But if we say 2 weeks*,* we may not be able to do it.*

*LF: I think we wouldn’t defer very much from these guys. After disclosure*,* there is that immediate one to know how they’re fairing. After the immediate follow-up*,* you do the two weeks then you give some space to monitor then after those two weeks depending on the changes that you have got then 4 weeks is okay. Because you also give them time to act out to think because they may be having questions in between*.

### Reorganization/paraphrasing

Participants highlighted that some aspects of the manual should be reorganized or paraphrased to ensure a consistent flow of interviews or for clarity of the intended information depending on the context. This is because some statements were deemed unclear or inappropriate and would be easily misinterpreted leading the interview in a different direction other than the intended.

*HCW: “No*,* it is not appropriate. Because if you mention any other health problems*,* they will tell us different other things not associated with HIV. Like an infection or sickle cell. Maybe you should ask them if they are affected by HIV. Maybe that would be better.”*

*LF: “I have a concern there*,* in situations where the mother may be negative*,* and the child is positive. That question does not apply to them. Yes*,* some children were given to grandparents to look after them.”*

## Discussion

The study aimed at adapting the clinic-based pediatric disclosure intervention developed in Ghana with task shifting from HCWs to CPSs in Eastern Uganda. The adaptation process included several changes related to sociocultural beliefs, norms, and perceptions, reorganizing/paraphrasing, boosting caregivers’ confidence to disclose, age appropriateness of content, disclosure mode, environment and person, and health system-related changes for the Ugandan setting.

HIV remains highly stigmatized in many settings; however, stigma is a sociocultural construct that is expressed differently across communities, hence the need to understand the unique factors influencing stigma in every context [37]. HIV-associated stigma at individual and community levels has been well documented as a major barrier to pediatric HIV status disclosure and must be considered while designing disclosure interventions for different settings [18, 38–40]. The changes made to this intervention address the specific Ugandan contextual beliefs, norms, and practices to ensure its relevance and appropriateness in the region. Additional changes aimed at boosting the caregivers’ confidence to disclose HIV status to the child were made. These included assessing the caregiver’s knowledge, fears, and disclosure readiness. These changes were meant to further address other known caregiver-related barriers to disclosure such as lack of disclosure knowledge, fears, or misinformation, which may vary across individuals and contexts [18, 39]. These barriers affect the readiness of caregivers to disclose and hence delay the initiation or completion of the HIV status disclosure process and affect the child’s engagement in care [20, 41]. As CPSs facilitate disclosure, it is important to understand both the context and individual-specific knowledge gaps and fears, to address them and assess the readiness of both the caregiver and the child before initiating the disclosure process.

In Uganda, as in many other African countries, extended and nuclear family types are common and many CLHIV are orphans raised by their relatives rather than their biological parents [2, 42, 43]. In such cases, several changes were made to ensure that the intervention manual considers the roles of other relatives who may not necessarily be HIV positive in the disclosure process, thereby boosting the child’s social support system.

Additionally, most Ugandan communities have long been conservative regarding sex education among children, with the responsibility mainly falling on adults. This has contributed to negative sexual health outcomes [44]. However, there are ongoing campaigns to encourage parents and caregivers of children to freely talk to their children about sexual and reproductive health to prevent undesirable outcomes like HIV infection or unplanned pregnancies [16, 45]. Hence, there is a need to carefully establish a middle ground for caregivers to decide when and how to deliver sex education to their children, which is an important component of pediatric HIV status disclosure.

Since HIV status disclosure is a process that should start at around age 5 (pre-school age) and be completed by age 12 (school-going age), the information provided should be age-appropriate and tailored to the child’s developmental stage and setting [10, 46]. To ensure clarity and understanding across several age groups, several terminologies and phrases related to HIV and status disclosure were modified accordingly.

Also, the mode and environment for status disclosure should be child-friendly, taking into account the child’s developmental stage and cognitive abilities to ensure good comprehension. Suggested modes for delivering disclosure information, such as role-playing and the use of media, books, and toys, align with disclosure interventions used in other settings. However, these interventions do not typically consider the involvement of multiple stakeholders [36, 47–49]. This highlights the importance of carefully selecting not only the content and information but also the mode of status disclosure and the roles of the various stakeholders involved in the process.

To ensure sustainability, several changes were made to the manual about the existing healthcare service delivery at the hospital. These changes included defining the identity of CPSs and the intervention and establishing a supervision, referral, and follow-up system for the intervention that conforms with that of the hospital. Research studies have shown that peer-supporter-based health interventions that align with existing healthcare structures are more cost-effective and sustainable, in both low-and middle-income and high-income settings [50–52]. In Uganda, the healthcare system already includes peer support services that operate under the supervision of specific healthcare workers for monitoring and referral [29, 53]. These peer supporters are also key in linking communities with hospitals and following up with clients in their communities. Therefore, the proposed CPS model was designed to fit seamlessly with the existing system.

Finally, several reorganizational changes were made, including paraphrasing certain terms to better align with the local context and ensure that CPSs could easily understand and implement the intervention. This was particularly important given that CPSs are laypersons rather than trained health professionals, for whom the manual was originally designed [36]. 

### Limitations

It is important to note that the steps we described here are only some of the steps of the ongoing adaptation process of the health intervention. Other steps to assess the appropriateness, feasibility, and acceptability of the adapted intervention will be ultimately be covered in the ongoing pilot testing. Additionally, efforts to obtain input from the intervention developers were unsuccessful, so we did not receive their input on the adaptation process.

## Conclusion

The findings from the adaptation process outlined here underscore the importance of engaging relevant stakeholders in adapting health interventions to ensure cultural appropriateness. While several health concepts may be similar across contexts, certain aspects can vary significantly based on individual, community, and health system-related factors. Therefore, this study sets an example of the adaptation process of an HIV disclosure intervention considering contextual and individual factors before rolling it out in a new setting. This approach is crucial to enhance the sociocultural appropriateness and uptake of the intervention.

## Electronic supplementary material

Below is the link to the electronic supplementary material.

**Supplementary Material 1**

**Supplementary Material 2**: **Appendix 1**

## Data Availability

Data is provided within the manuscript or supplementary information files.
