# Supplementary material for: Cultural adaptation of clinic-based pediatric hiv status disclosure intervention with task shifting in Eastern Uganda
Source: AIDS Res Ther. 2025 Apr 19;22:48. doi: 10.1186/s12981-025-00743-7 (PMC12008972; doi:10.1186/s12981-025-00743-7)
Supplement: Supplementary file 1 — Supplementary Material 1 [file 12981_2025_743_MOESM1_ESM.docx]

**Adapted Pediatric HIV Disclosure Manual**

This manual was developed to guide the delivery of an HIV pediatric disclosure intervention. The intervention is guided by a theory and empirically- guided disclosure model, best practice recommendations of the American Pediatric Association, the Thai Pediatric HIV Disclosure Manual (2009), clinical experiences and research conducted by the authors and others.

**Table of contents**

Contents

[Introduction 3](#_Toc172099437)

[Conceptual Background & rationale 4](#_Toc172099438)

[Overview Intervention 7](#_Toc172099439)

[Pre-Disclosure: Session I 9](#_Toc172099440)

[Step 1. Assess dimensions of the caregiver’s disclosure knowledge, motivation and behavioral skills. 9](#_Toc172099443)

[Step 2. Assess caregiver responses to the questions. 10](#_Toc172099444)

[Step 3. Set up conditions for perceptual change. 11](#_Toc172099445)

[Step 4. Introduce replacement perceptions and alternate behaviors. 11](#_Toc172099446)

[Step 5. Conclude visit 12](#_Toc172099447)

[Follow-up Sessions: Pre-Disclosure (2-weekly sessions) 13](#_Toc172099448)

[Key Information, Behavior/Skills, Affective Support Content for Caregivers 14](#_Toc172099449)

[Disclosure Sessions 15](#_Toc172099450)

[Summary 15](#_Toc172099451)

[Step 1. Assess the child’s readiness. 15](#_Toc172099452)

[Step 2. Make a specific plan. 16](#_Toc172099453)

[Step 3. Follow up. 17](#_Toc172099454)

[Post-disclosure Sessions 17](#_Toc172099455)

[Summary 17](#_Toc172099456)

[Appendices 18](#_Toc172099457)

[Appendix 1: HIV status disclosure questionnaire 18](#_Toc172099458)

[References 19](#_Toc172099459)

# Introduction

The scale-up of antiretroviral therapy (ART) in resource-limited settings is dramatically changing the landscape of pediatric HIV care. Millions of children infected with HIV at birth now have access to life-saving therapy and are expected to live into adulthood. Yet, expanded access to ART is not, in and of itself, sufficient for these expectations to be fully realized. Changes in other inter-related elements of pediatric care are also needed; among these, informing the child that s/he has HIV in a timely, age-appropriate manner. The process of disclosing an HIV diagnosis to the child has been recommended by leaders in the field for over a decade ^1^,^2^. This recommendation is consistent with child rights and research showing that it confers several benefits, importantly, better adherence to therapy, better clinical outcomes, better psychological adjustment and lower risk of transmitting HIV when the child becomes sexually active. However, it is estimated that as many as 75% of children in some regions have not been informed that they have HIV. This challenging and pervasive problem is even more critical in the era when children have the opportunity to survive childhood and live through adolescence.

This intervention is grounded in a traditional Ghanaian concept, “SANKOFA”, and behavioral and bioecological systems theory. The patient-centered intervention approach uses an Adherence and Disclosure specialist model where a designated specialist familiar with the socio-cultural norms of the community is well trained to target modifiable information, motivation and behavioral skills of caregivers to facilitate their engagement in the process of disclosure (i.e., pre-disclosure, disclosure, and post-disclosure phases) in a manner suitable to the needs of the child.

## Conceptual Background & rationale

“The intervention is grounded in the principles of Sankofa and guided by an HIV pediatric disclosure model drawn from our prior research conducted in Ghana and that of others, bioecological systems theory, and core elements of the Information-Motivation-Behavioral Skills (IMB) model of Health Behavior Change ^18^ and other applications of it ^19,^ ^20^.The model applies these conceptualizations to a dynamic behavior that requires specific attention to multiple

systems that simultaneously influence an individual’s decisions to disclose HIV status to the child. The specific content of the core IMB-areas (information, motivation, behavioral skills) is articulated to the process of initiating and sustaining disclosure behavior over time (Fig. 1). With the integration of bioecological systems theory, emphasis is placed on situating the core variables within the cognitive-affective, socio-cultural environment, developmental age of the HIV-infected child, and systems of relationships that form the caregiver’s and child’s environment in which disclosure occurs.

Consistent with the essence of the IMB model, the interrelations among the core constructs of the disclosure model specify that disclosure information and disclosure motivation most often work through disclosure behavioral skills to affect the caregiver’s process of HIV status disclosure (or non-disclosure) to the child. ***Information*** that is highly relevant to disclosure may be accurate information or faulty heuristics or misinformation that the caregiver has about disclosure of HIV status to the child. Critical to disclosure is accurate information about HIV treatment and prognosis, why adherence is important and relationship to disclosure, age/ development appropriate-content to inform the child about HIV status and what to expect of the child’s reaction and adjustment and sources of support. ***Motivation*** is the personal and social beliefs regarding the positive and negative consequences of disclosure and non-disclosure.

These are influenced by past experiences and socio-cultural milieu. For example, this would

include the caregiver’s perception of stigma and perceptions of consequences if others learn of the child’s HIV status. Prior research has demonstrated the importance of perceived benefits outweighing negative consequences in the individual’s decision to disclose ^4,^ ^5,^ ^7^. ***Behavioral Skills*** are objective and perceived skills and one’s sense of self-efficacy in implementing these skills requisite for effective disclosure of HIV status. These include skills to communicate effectively with the child about his/her diagnosis in an age appropriate manner and activate sources of support to enhance child’s adjustment. Information, motivation and behavioral skills influence disclosure behavior which in turn influences the child’s health outcomes (e.g., adherence, virologic). ***Disclosure*** is understood as a process with caregivers moving through phases of pre-disclosure, disclosure, and post-disclosure. Disclosure of HIV status to the child is not a one-time event, but rather a process, involving ongoing discussions about the disease as the child matures cognitively, emotionally, and sexually. The child is viewed from a developmental perspective within the context of his or her environment and systems of relationships. Children’s perception of self, health, illness, and death evolve as they mature through different developmental stages. The interaction between factors in the child’s maturing biology, his immediate family/community environment, and the societal landscape influences his/her development. Once the diagnosis has been explained to a child, it needs to be reinforced or regularly discussed as the child develops because many children will not have understood the full implications of the disease or diagnosis at the time of disclosure ^21,^ ^22^. For example, preadolescent children can cognitively understand the concepts about the virus but may be less likely to think of the future implications, such as transmission risks and safe sexual practices.

Correlates of disclosure identified in our prior work and that of others map well onto the IMB constructs; hypothesize that disclosure will be more likely when well informed about HIV and perceives benefits of disclosure, positive/supportive relationships and have the skills to engage in the disclosure process. For example, in studies conducted in Zambia and the Democratic Republic of the Congo, a common reason caregivers gave for disclosure is the hope that by knowing their status, the children will have better adherence to treatment ^6,^ ^15^. The role of cognitive-affective processes in terms of acceptance of one’s and/or child’s HIV have been recently highlighted as well**.** Further, as we have found in our work in Ghana, and as others have also observed, for some caregivers, navigation through the stages may be difficult if the

caregiver lacks disclosure skills and most often will require or expect medical professionals to help them through the process ^2,^ ^13,^ ^23-25^.

**Fig.1. Conceptual Disclosure Framework**


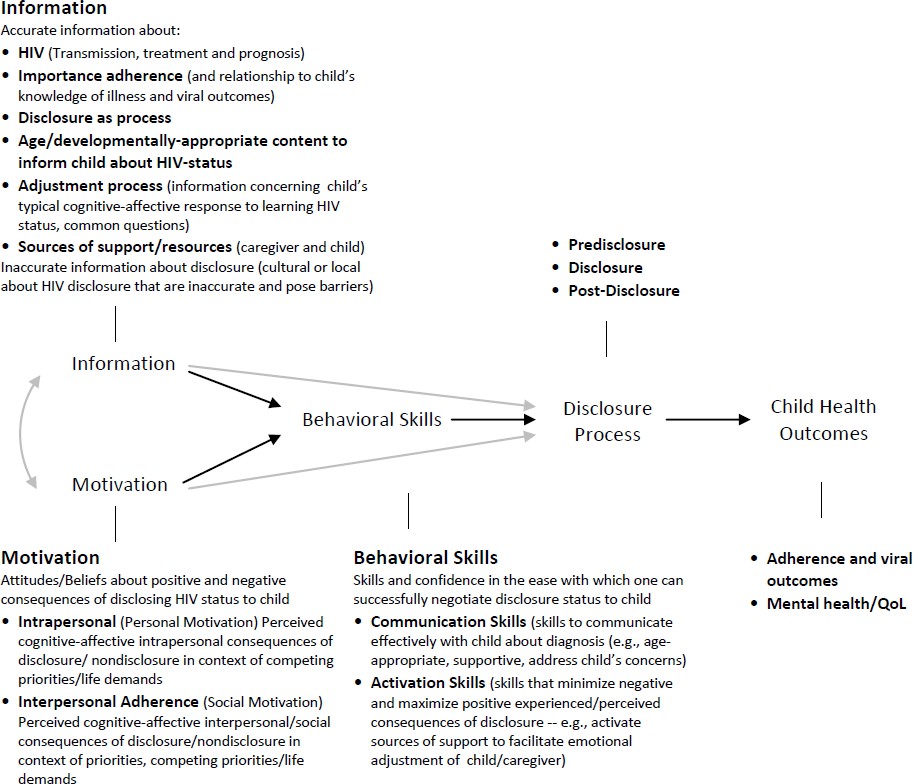


# Overview Intervention

The intervention is guided by the disclosure model and contains two key elements to target well- documented, modifiable barriers to promote disclosure.

- The first component is use of an adherence and disclosure **specialist model** where a designated specialist (hitherto, referred to as the “CPS”) familiar with the socio-cultural norms of the community is well trained to assist families go through the process of disclosure (i.e., pre-disclosure, disclosure, and post-disclosure phases).

Our preliminary work has shown that caregivers have prefer to have assistance from a health care provider. Further, a specialist (as opposed to team) in the clinic will be used because of the cultural environment and complexity of disclosure process that makes having specific point person advisable. This will allow continuity and foster development of a trusting relationship with the caregiver and child.

- The second component is **disclosure as a process** whereby the CPS will guide the intervention sessions to the Information, Motivation and Behavioral Skills needs of the caregiver and the age and neurocognitive development of the child. There will be scheduled education and counseling sessions, divided into three main phases: pre- disclosure, disclosure, and post-disclosure as outlined in the HIV disclosure intervention schema (Figure 5).

A summary of key issues^33^ to be discussed during these sessions are outlined in Table

1. The CPS will have frequent and scheduled sessions with eligible caregiver and child dyads. The pre-disclosure sessions will address the caregiver’s disclosure information, motivation and behavioral skills. During these individualized sessions, the caregiver will learn the benefits of disclosure, how to disclose to a child in developmentally-appropriate manner, adjustment and coping after disclosure. The caregiver’s concerns about negative consequences of disclosure will be discussed and coping skills, social problem solving, and communication skills developed with didactic and interactive techniques. For example, the CPS will have a mock disclosure session with the caregiver where all

the difficult questions anticipated from the child will be discussed. The date and venue of the disclosure will be discussed as well. If the caregiver chooses to disclose at home or the opportunity avails, the CPS will provide a phone number for the caregiver to call after the event to register the exact date of disclosure and also provide support to the caregiver if needed. If the caregiver chooses to have the CPS present at disclosure the venue and date will be discussed. After disclosure, the CPS will continue to meet with the caregiver and child at frequent and scheduled intervals to assess post-disclosure problems and coping, provide referral to services that the family may need, continue to help the child to understand in an age-appropriate manner HIV infection and its implication on his/her day-to-day activities.

**Table 1. Summary Disclosure Process**

11

| **Pre-Disclosure Sessions**  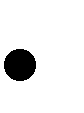 Meeting of the Adherence Disclosure Specialist (CPS) with the parent/caregiver to start the process.  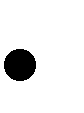 Discuss importance of disclosure and ascertain whether the family has a plan in mind.  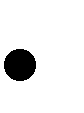 CPS to respect and be sensitive to the intense feelings of the caregiver about disclosure.  Access anticipated response of the child if he/she is told of the diagnosis 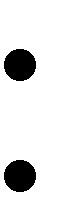 If the caregiver is ready to disclose, guide them in various ways of approaching disclosure.  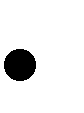 If the caregiver is not ready to disclose, schedule follow-up meeting to discuss the need for disclosure, how to disclose, the support system in place for and after disclosure; provide reading materials to help with the process.  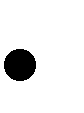 Respect the caregivers timing, but strongly, encourage the caregiver not to lie to the child about the diagnosis |
| --- |
| **Disclosure Sessions**  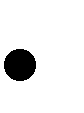 When the caregiver is ready, have him/her think through or write out how they want the conversation to go.  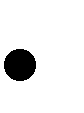 Caregiver should choose a place where the child will be most comfortable to talk openly.  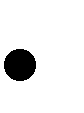 The CPS will provide the caregiver with questions that the child may ask so the caregiver is prepared with open and honest answers.  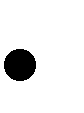 If the caregiver cannot do this alone, the CPS may offer to be present at disclosure.  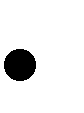 At disclosure, medical facts should be kept to the minimum. The child needs to hear that they didn’t do or say anything to cause the disease and that the family will always support him/her.  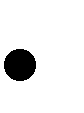 The CPS will offer to give the family a call during the week and schedule a follow- up meeting two weeks after disclosure. |
| **Post-disclosure Sessions**  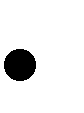 Follow-up sessions with the family at 2 weeks, 4 weeks, and every 2 to 4 weeks for the first six months to assess impact of disclosure, to answer questions, and to help foster support between the child and the family.  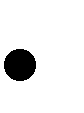 At these meetings, ask the child to tell you what they have learnt about the virus.  This will provide the opportunity to clarify any misconceptions. Assess changes in emotional well-being. 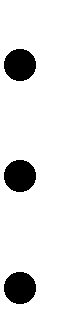 Support caregiver for having disclosed the diagnosis.  Remind parents that disclosure is not one time event. |

**I. Pre-Disclosure Sessions**

**Summary**

- Meeting of the CPS with the parent/caregiver to start the process.
- Discuss importance of disclosure and ascertain whether the family has a plan in mind.
- CPS to respect and be sensitive to the intense feelings of the caregiver about disclosure.
- Access anticipated response of the child if he/she is told of the diagnosis
- If the caregiver is ready to disclose, guide them in various ways of approaching disclosure.
- If the caregiver is not ready to disclose, schedule follow-up meeting to discuss the need for disclosure, how to disclose, the support system in place for and after disclosure; provide reading materials to help with the process.
- Respect the caregivers timing, but strongly, encourage the caregiver not to lie to the child about the diagnosis

# Pre-Disclosure: Session I

The CPS will spend approximately 45 minutes with the caregiver using the recommended strategies. Session I and each follow-up session will be adapted so that it is individualized to the caregiver but will stay true to the disclosure model protocol and motivational interviewing techniques (Miller and Rollnick).

## Greet caregiver, state purpose of meeting and begin to establish rapport. Example:

“Hello *name of client*. My name is . I work as CPS at Jinja RRH. I understand that you have agreed to participate in this study. Is that correct? It’s nice to meet you. I will be meeting with you and will be available to assist you and answer questions you have about your child’s HIV status and what to discuss with him/her about it. Do you have questions about this?”

## “I’d like to get to know a little about you and (child’s name). Do you mind if I ask you a few questions?”


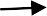

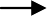


If the caregiver **does not** agree: Seek clarification.

- If it is because it is not a good time for the caregiver to talk, reschedule the visit. If it is because the caregiver is ambivalent or reluctant to participate in the intervention, begin to explore further using MI techniques as appropriate.
- If the caregiver does not wish to proceed at this time, but is willing to at another time, reschedule visit. Remind caregiver that s/he also has the option to call you [per study parameters] as desired.
- If other reasons specify
- If caregiver is willing to continue after clarification, proceed to baseline assessment of child’s illness and caregiver’s disclosure knowledge, motivation and behavioral skills (Step 1).

If the participant **does agree**: Begin baseline assessment of caregiver HIV illness/disclosure beliefs (Step 1).

## Step 1. Assess dimensions of the caregiver’s disclosure knowledge, motivation and behavioral skills.

Use a conversational approach. Assess the caregiver and child’s current circumstances, family situation, caregiver support system, concerns, knowledge and attitudes about disclosure. Questions do not have to be asked in a particular order. Respond in ways that encourage caregivers to explain their circumstances in detail. Listen carefully.

- “I’d like to ask you about (name of child). How is his/her health?
- “Has (child’s name) ever had difficulties taking his/her HIV medicines as ordered? If yes, What happened?”
- “Are you affected by your child’s HIV status? Is anyone else in the family having a similar illness?”
- “Who else knows about (child’s name) HIV status?”
- “Has (child’s name) ever asked you about his/her illness or reason for taking medicines?”
- “Have you ever talked to (child’s name) about his/her HIV status?”
- “Have you considered discussing (child’s name) HIV status with him/her?” if no, why?
- “In your opinion, are there any reasons that it would be unwise to discuss (child’s name) HIV status with him/her at this time?” “Do you have any particular concerns?”
- “On a scale of 1-10, how important would you say is it for (Child’s name) to know about his/her HIV status?” **1=not at all important and 10=extremely important**
- “How confident would you say you are that you are about discussing this with him/her?”

**1=not at all confident or 10=extremely confident**

## Step 2. Assess caregiver responses to the questions.

Identify disclosure informational, motivational and skills perceptions that may an impediment to disclosure. This will allow you to intervene in a way that best addresses the caregiver’s needs.

**Examples of Common Barriers to Disclosure:**

- - **Informational**
    - **Articulates poor understanding or has inaccurate information regarding:**
      - Age/developmentally appropriate content to inform child about HIV-status information (e.g., mode of HIV transmission, treatment and prognosis)
      - Importance of adherence (and relationship to child’s knowledge of illness and viral outcomes)
      - Disclosure as process adapted to child’s age and level of understanding
      - Adjustment process (information concerning child’s typical cognitive-affective response to learning HIV status, common questions)
      - Sources of support/resources (caregiver and child)
  - **Motivational**
    - **Articulates a**ttitudes/beliefs about negative consequences of disclosing HIV status to child
      - **Intrapersonal** (Personal Motivation) --Perceived negative intrapersonal consequences of disclosure/ nondisclosure in context of competing priorities/life demands
      - **Interpersonal Adherence** (Social Motivation) Perceived negative interpersonal/social consequences of disclosure/nondisclosure in context of priorities, competing priorities/life demands.
  - **Behavioral Skills**
    - Demonstrates lack of confidence in skills with which caregiver can successfully negotiate informing child of his/her HIV status.
      - Expresses low confidence in ability to communicate effectively with child about diagnosis (e.g., age-appropriate, supportive, address child’s concerns) without upsetting him/her.
      - Activation Skills -- skills that minimize negative and maximize positive experienced/perceived consequences of disclosure -- e.g., activate sources of support to facilitate emotional adjustment of child/caregiver.

## Step 3. Set up conditions for perceptual change.

Explore caregiver perceptions that may impede disclosure. Encourage the caregiver to think about illness experiences, interactions with others, sources of information, cognitive and emotional processes that contributed to the perceptions.

Example:

“You said that you think that telling your child that he has HIV is too risky. Can you explain why you feel this way?”

Encourage the caregiver to discuss how he/she has attempted to manage the experiences.

Example:

“It sounds like you are worried that your child is too young to know of his diagnosis. Have you ever considered telling him?”

**Discuss the strategies used and beliefs about benefits and limitations.**

Example:

“Do you think not telling your child that he has HIV is the best strategy? How does that work for you? Do you see any problems with noy telling him? Have you considered telling him in a way geared to his age and level of understanding?

**Evaluate caregiver responses.**


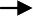

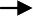


If caregiver expresses lack of satisfaction or limitations of non-disclsoure, begin to introduce replacement perceptions (Step 5).

If caregiver expresses fatality, resistance to considering alternate approaches explore further using MI techniques.

## Step 4. Introduce replacement perceptions and alternate behaviors.

Present key information, motivational, behaviors/skills and affective support content individualized to the caregiver’s perceptual system and his/her context. It is important to focus on areas that have been identified as possible impediments to disclosure.

However, the CPS may ask if the caregiver perceives that he/she has any questions or interest in learning more about key content.

To the greatest extent possible, use language and rationale that conform to the caregiver’s level of understanding and vocabulary. Avoid use of medical jargon unless patient demonstrates a high level of health literacy.

Give examples using information learned about the patient in the baseline assessment.

Use a friendly, empathetic, NON-CONFRONTATIONAL approach. The CPS should reinforce the caregiver as problem-solver with the CPS as facilitator.

Return to Step 4 and use MI strategies accordingly if responses indicate resistance.

## Step 5. Conclude visit

- 1. **Summarize the topics that have been discussed.**


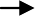

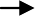


**Intention or optimism for disclosure (e.g., caregiver begins to ask questions or seek information about strategies, express commitment to “do better”, or less resistant to trying something new).**

**Reinforce positive commitment or move toward positive changes. Make appointment to proceed to the next step (Disclosure)**

Example:

“It sounds like you might be ready to try…” “Would you like more information about this?”

“I hear you sounding like you might be ready to inform your child of his diagnosis. Is that true?/Would you agree?”

**Caregiver sees problems/resistant to change perceptions/behaviors that may impede effective disclosure.**

**Be supportive. Normalize their concerns/feelings**. Example:

“So, here’s what I’m hearing you say so far: On one hand you……and on the other you….”

“Many people have similar concerns when they are thinking about informing a child of his/her HIV diagnosis.”

- 1. **Inquire if caregiver has any questions and answer them.**
  2. **Inform caregiver when you will be contacting him/her again and how he/she can reach you in interim.**

# Follow-up Sessions: Pre-Disclosure (2-weekly sessions)

1. Create a relaxed and friendly atmosphere. Inquire about the caregiver’s and child’s well-being since the last contact. Review what was discussed in last visit and inquire whether the caregiver has given informing child of his/her HIV status any further thought. Example:

“At your last visit, we discussed informing your child of his/her HIV status. May I ask what you thought that day?


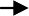

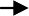


If caregiver reports that he/she has been doing well and expresses interest in disclosing HIV diagnosis to child, proceed to Disclosure protocol.

If caregiver reports that he/she has concerns about disclosing, repeat Steps 2-6 focusing on areas of concern or proceed to b as appropriate.

1. Inquire if the caregiver has any questions and answer them.
2. Arrange time to meet with caregiver again and remind him/her how you can be contacted in the interim.

The caregiver is always the one who determines whether or not the caregiver and/or child is ready. The CPS only facilitates the caregiver’s reflection and provides information to facilitate the decision-making process.

## Key Information, Behavior/Skills, Affective Support Content for Caregivers

**Why tell your child about HIV? There are lots of good reasons**

- You want your child to hear it from you
- You want to be open and honest
- Keeping secrets is hard
- Children often know something is wrong
- Children can have amazing ability to deal with the truth
- You want to help your child feel more in control
- You want your child to understand why they have to take medications and go to the hospital to have blood drawn and
- It’s their right to know
- Protecting others.

**HIV modes of transmission, difference between HIV and AIDS, importance of treatment How will child react to knowledge about his/her illness?**

- Children are all different. Think about how your child reacts to other things. This may give you some ideas about what to expect.

**Sources of support**

- Finding friends who provide encouragement and support
- Engaging support from others

**How can caregiver support my child?**

**What should I do if my child becomes upset? Disclosure/stigma/secrecy:**

- Deciding who to tell
- Ways to help child keep diagnosis private

**Remembering regimen:**

- Strategies for remembering to take medications (e.g., incorporating medications into daily routine)
- Ways of adapting to changes in schedule and environment (e.g., weekends, trips)
- Safety plans (e.g., taking drugs when going out at night if may not return before AM dose)

# II. Disclosure Sessions

## Summary


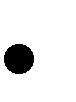
 When the caregiver is ready, have him/her think through or write out how they want the conversation to go.


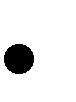
 Caregiver should choose a place where the child will be most comfortable to talk openly.


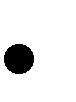
 The CPS will provide the caregiver with questions that the child may ask so the caregiver is prepared with open and honest answers.


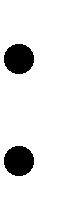
If the caregiver cannot do this alone, the CPS may offer to be present at disclosure.

At disclosure, medical facts should be kept to the minimum. The child needs to hear that they didn’t do or say anything to cause the disease and that the family will always support him/her.

When the caregiver has decided that she/he would like the child to be informed of her/his HIV status, the CPS will focus on how to prepare the caregiver and child.

## Step 1. Assess the child’s readiness.

The CPS will meet with the child to build rapport and trust assess his/her readiness. Examples: “I’m so glad to have a chance to talk to you today.”

“How old are you now?”

“What kinds of things do you like to do the most?”

The CPS will ask questions in a relaxed way without putting pressure on the child. It is helpful to start by asking younger children to draw a picture.

The CPS will begin encouraging the child to verbalize their thoughts about more difficult topics. Examples:

“Can you tell me about your school? How are things for you there? What do you usually do at school during your lunch break? Do you ever have any problems? How do you handle those?”

“You say your friends sometimes tease you. What do you do when that happens? Does it work?’

“When you are worried about something, who do you talk to? Are you comfortable asking them questions about anything?”

**Readiness of the Child or Adolescent** (Glaser, 2016)

Review what the child understands about their own health.

Assess clinical status to confirm that the patient does not have severe illness.

Find out what the child or adolescent knows about the medicines he/she has been taking and about their illness.

Review the patient’s mood and behavior.

Review with the child how they cope with stressful situations.

Review the child’s ability to keep a secret or maintain confidentiality.

Find out the patient’s relationship with peers in school or at home.

Note: CPS should complete the Pediatric Disclosure Readiness Assessment Checklist (appendix 1)

The CPS should avoid directly discussing the child’s HIV diagnosis at this point, but some children may show that they are suspicious or already know of the HIV diagnosis. In such cases, the CPS should probe further to find out what the child has learned and how she/he is feeling. If the child has any frightening or negative perceptions, the CPS should provide the correct information to them.

## Step 2. Make a specific plan.

After the caregivers and child have been assessed and found ready, and the caregiver has decided the child should be informed, the caregiver should prepare by making a specific plan. The caregiver should decide where and whether they want to disclose to the child him/herself or whether they would prefer for the CPS assist with the disclosure.

If the caregiver decides to disclose at home, the CPS will review and rehearse how the child will be informed and how she/he will answer questions that the child may ask. The CPS will provide the caregiver with age/developmentally appropriate information and responses and role play the session.

The plan should include the following:

- Selection of a time when the child is physically, emotionally and psychologically healthy and not under stress (e.g., birthday).
- Plan appropriate approach for informing the child, based on their age/maturity and specific needs. Different media may be used such as story book or illustrations.
- Prepare the content of the discussion, covering the following points:
  - Tell the child he/she has HIV
  - Give information about HIV
  - Explain the difference between HIV and AIDS
  - Explain how to care for themselves and prevent further transmission
  - Discuss how to keep a secret
  - For adolescents or children at risk for unsafe sex, the caregiver decides whether or not to tell them about how to practice safe sex
  - Other information according to age and the interest of the child
  - Rehearse the caregiver’s answers to anticipated questions, especially sensitive questions such as how the child acquired HIV
  - Consider how the caregiver will respond if the child is upset at the time the child is informed of her diagnosis (e.g., frightened or angry).
  - Provide the caregiver with the CPS emergency contact number and a back- up emergency number of the child’s health care provider.

Examples:

“According to your plan, when will you talk to your child?” “How will you start the conversation with your child?”

“What information do you want or don’t you want your child to know?”

“What will you do if your child becomes sad or starts crying after you tell him/her of the diagnosis?”

“How will you respond if the child asks how she/he got HIV?”

## Step 3. Follow up.

The CPS will offer to give the caregiver during the week and will schedule a follow-up meeting with the caregiver and child soon after the disclosure at home. This will provide the child with the opportunity to ask questions about things they did not understand well and for the CPS to monitor how the child is adjusting to the information.

# III. Post-disclosure Sessions

## Summary


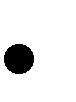
 Follow-up sessions with the family immediately (within 3 days) after disclosure, at 2 weeks, then 4 weekly, for the first six months to assess impact of disclosure, to answer questions, and to help foster support between the child and the family.

Note: This can be done through phone calls or face to face meetings as per the convenience of clients and CPS.


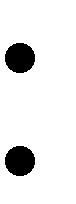

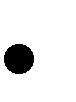
 At these meetings, ask the child to tell you what they have learnt about the virus. This will provide the opportunity to clarify any misconceptions.

Assess changes in emotional well-being.

Support caregiver for having disclosed the diagnosis.

The CPS will have follow-up sessions with the caregiver and child after the child has been told of his/her diagnosis. Follow-up sessions will be at 2 weeks, 4 weeks, and every 2 to 4 weeks for the first six months to assess impact of disclosure, to answer questions, and to help foster support between the child and the family.

**Step 1. Explain reason for session.**

The CPS will begin the session using same counseling techniques as used in previous steps. The caregiver will state objectives and obtain agreement from the caregiver and child before starting. Example:

“The last time we met, we talked a lot about your health. The appointment today is to review all of the things we talked about at your last visit.”

**Step 2. Assess, review and answer child’s questions**

Review topics discussed at the last meeting and provide additional information depending on child’s needs/questions.

- Assess the child’s understanding of HIV and modes of transmission.
- Assess how the child keeps secrets and whether the child has shared his diagnosis with anyone? Example:

“After you learned about your HIV, did you tell anyone?”

“What would you say if someone asked you about your HIV or why you take medication?

- Assess any behavior changes and adaptations in physical, emotional, and social health.
- Assess child’s adherence to treatment

**Step 3. Assess, review and answer caregiver’s questions.**

Assess caregiver’s view of how child has been adjusting learning she/he has HIV. Address concerns and answer questions. Make referrals as necessary to the social worker

Remind parents that disclosure is not one time event and how the child’s informational and social support needs will change and need to be addressed over time.

# Appendices

## Appendix 1: Pediatric Disclosure Readiness Assessment Checklist

| **Task 1. Assess the child for disclosure eligibility** | Date task 1 executed: | | Task Facilitator’s Name: |
| --- | --- | --- | --- |
| Child has met the age criteria (between 6 and 10 years) | Yes | No |  |
| Child and caregiver knowledge on benefits of disclosure | Yes | No |  |
| Caregiver willing to disclosure to child | Yes | No |  |
| Task 1 Comments: | | | |
| **Task 2. Assess the child and caregiver readiness** | Date task 2 executed: | | Task Facilitator’s Name: |
| Child and caregiver free from severe physical illness, trauma, psychological illness, or psychiatric illness? | Yes | No |  |
| Child has consistent family, peer support or social support | Yes | No |  |
| Child demonstrates interest in the environment and playing activities | Yes | No |  |
| Child already knows about the medicines and illness and addressed needs and concerns | Yes | No |  |
| Functional school engagement by the child (consistent attendance, interacts well with the school community, able to freely discuss school activities) | Yes | No |  |
| Caregiver ready for disclosure to the child | Yes | No |  |
| Caregiver has communicated with the child to assess readiness | Yes | No |  |
| Management of confidentiality of information regarding one’s health discussed with the child and caregiver | Yes | No |  |
| Task 2 Comments: | | | |

Adopted form the Disclosure of HIV Status Toolkit for Pediatric and Adolescent Populations(Glaser, 2016)

# References

Bachanas PJ, Kullgren KA, Schwartz KS, et al. Predictors of psychological adjustment in school-age children infected with HIV. *J Pediatr Psychol.* Sep 2001;26(6):343-352.

Blasini I, Chantry C, Cruz C, et al. Disclosure model for pediatric patients living with HIV in Puerto Rico: design, implementation, and evaluation. *J Dev Behav Pediatr.* Jun 2004;25(3):181-189.

Boyd-Fanklin N AJ, Steiner G, Drelich E, Norford B. Family systems interventions and family therapy. In: Gloria Steiner MB, ed. *In: Children, Families and HIV/AIDS*; 1995:115-126.

Britt E, Hudson SM, Blampied NM. Motivational interviewing in health settings: a review. *Patient Educ Couns.* May 2004;53(2):147-155.

Butler AM, Williams PL, Howland LC, Storm D, Hutton N, Seage GR, 3rd. Impact of disclosure of HIV infection on health-related quality of life among children and adolescents with HIV infection. *Pediatrics.* Mar 2009;123(3):935-943.

Disclosure of illness status to children and adolescents with HIV infection. American Academy of Pediatrics Committee on Pediatrics AIDS. *Pediatrics.* Jan 1999;103(1):164-166.

Disclosure of HIV to Perinatally Infected Children and Adolescents: The New York State Department of Health AIDS Institute HIV Clinical Guidelines November 2009.

Ferris M, Burau K, Schweitzer AM, et al. The influence of disclosure of HIV diagnosis on time to disease progression in a cohort of Romanian children and teens. *AIDS Care.* Oct 2007;19(9):1088-1094.

Fisher JD, Fisher WA. Changing AIDS-risk behavior. *Psychol Bull.* May 1992;111(3):455-474.

Fisher JD, Amico KR, Fisher WA, Harman JJ. The information-motivation-behavioral skills model of antiretroviral adherence and its applications. *Curr HIV/AIDS Rep.* Nov 2008;5(4):193-203.

Funck-Brentano I, Costagliola D, Seibel N, Straub E, Tardieu M, Blanche S. Patterns of disclosure and perceptions of the human immunodeficiency virus in infected elementary school-age children. *Arch Pediatr Adolesc Med.* Oct 1997;151(10):978-985.

Glaser, E., 2016. Disclosure of HIV Status Toolkit for Pediatric and Adolescent Populations.

Hardy MS, Armstrong FD, Routh DK, Albrecht J, Davis J. Coping and communication among parents and children with human immunodeficiency virus and cancer. *J Dev Behav Pediatr.* Jun 1994;15(3 Suppl):S49-53.

Harries AD, Gomani P, Teck R, et al. Monitoring the response to antiretroviral therapy in resource-poor settings: the Malawi model. *Trans R Soc Trop Med Hyg.* Dec 2004;98(12):695-701.

Kallem S, Renner L, Ghebremichael M, Paintsil E. Prevalence and pattern of disclosure of HIV status in HIV-infected children in Ghana. *AIDS Behav.* Aug;15(6):1121-1127.

Lee CL, Johann-Liang R. Disclosure of the diagnosis of HIV/AIDS to children born of HIV- infected mothers. *AIDS Patient Care STDS.* Jan 1999;13(1):41-45.

Lesch A, Swartz L, Kagee A, et al. Paediatric HIV/AIDS disclosure: towards a developmental and process-oriented approach. *AIDS Care.* Jul 2007;19(6):811-816.

Lester P, Chesney M, Cooke M, et al. When the time comes to talk about HIV: factors associated with diagnostic disclosure and emotional distress in HIV-infected children. *J Acquir Immune Defic Syndr.* Nov 1 2002;31(3):309-317.

Lipson M. What do you say to a child with AIDS? *Hastings Cent Rep.* Mar-Apr 1993;23(2):6-12.

Mellins CA B-CE, Dolezal C, Richards A, Nicholas SW, Abrams EJ. Patterns of HIV status disclosure to perinatally HIV-infected children and subsequent mental health outcomes. *Clin Child Psychol Psychiatry.* 2002;7:101-114.

Menon A, Glazebrook C, Campain N, Ngoma M. Mental health and disclosure of HIV status in Zambian adolescents with HIV infection: implications for peer-support programs. *J Acquir Immune Defic Syndr.* Nov 1 2007;46(3):349-354.

Mills EJ, Nachega JB, Buchan I, et al. Adherence to antiretroviral therapy in sub-Saharan Africa and North America: a meta-analysis. *Jama.* Aug 9 2006;296(6):679-690.

Nabukeera-Barungi N, Kalyesubula I, Kekitiinwa A, Byakika-Tusiime J, Musoke P. Adherence to antiretroviral therapy in children attending Mulago Hospital, Kampala. *Ann Trop Paediatr.* Jun 2007;27(2):123-131.

Nehring WM, Lashley FR, Malm K. Disclosing the diagnosis of pediatric HIV infection: mothers' views. *J Soc Pediatr Nurs.* Jan-Mar 2000;5(1):5-14.

Oberdorfer P, Puthanakit T, Louthrenoo O, Charnsil C, Sirisanthana V, Sirisanthana T. Disclosure of HIV/AIDS diagnosis to HIV-infected children in Thailand. *J Paediatr Child Health.* May 2006;42(5):283-288.

Report on the global AIDS epidemic; executive summary. [http://data.unaids.org/pub/GlobalReport/2008/JC1511_GR08_ExecutiveSummary_en.pd](http://data.unaids.org/pub/GlobalReport/2008/JC1511_GR08_ExecutiveSummary_en.pdf.%20Accessed%209/13/2008)

[f. Accessed 9/13/2008](http://data.unaids.org/pub/GlobalReport/2008/JC1511_GR08_ExecutiveSummary_en.pdf.%20Accessed%209/13/2008).

Rivet Amico K. A situated-Information Motivation Behavioral Skills Model of Care Initiation and Maintenance (sIMB-CIM): an IMB model based approach to understanding and intervening in engagement in care for chronic medical conditions. *J Health Psychol.*

Oct;16(7):1071-1081.

Schonfeld DJ. Talking with elementary school-age children about AIDS and death: principles and guidelines for school nurses. *J Sch Nurs.* Feb 1996;12(1):26-32.

Vaz L, Corneli A, Dulyx J, et al. The process of HIV status disclosure to HIV-positive youth in Kinshasa, Democratic Republic of the Congo. *AIDS Care.* Aug 2008;20(7):842-852.

Vreeman RC, Nyandiko WM, Ayaya SO, Walumbe EG, Marrero DG, Inui TS. The perceived impact of disclosure of pediatric HIV status on pediatric antiretroviral therapy adherence, child well-being, and social relationships in a resource-limited setting. *AIDS Patient Care STDS.* Oct;24(10):639-649.

Wiener L. HIV Disclosure. In: Zeichner SL R, JS, ed. *Textbook of Pediatric HIV Care*: Cambridge University Press; 2005.

Weiner L. *Helping a parent with HIV tell his or her children*. Binghamton, NY: Haworth Press; 1998.

Wiener L, Mellins CA, Marhefka S, Battles HB. Disclosure of an HIV diagnosis to children: history, current research, and future directions. *J Dev Behav Pediatr.* Apr 2007;28(2):155-166.
