# Supplementary material for: Cultural adaptation of clinic-based pediatric hiv status disclosure intervention with task shifting in Eastern Uganda
Source: AIDS Res Ther. 2025 Apr 19;22:48. doi: 10.1186/s12981-025-00743-7 (PMC12008972; doi:10.1186/s12981-025-00743-7)
Supplement: Supplementary file 2 — Supplementary Material 2: Appendix 1 [file 12981_2025_743_MOESM2_ESM.docx]

**Appendix 1**

|  |  |  |
| --- | --- | --- |

Table 3. **A summary of changes between the original and adapted versions of the pediatric HIV disclosure intervention manual.**

| Item/Change | Original version | Adapted version |
| --- | --- | --- |
| **General changes** |  |  |
| - Person delivering the intervention | Adherence and disclosure specialist (ADDS) | Caregiver Peer Supporter |
| - Name | “*Project Sankofa*” | Replaced with “*this study*” |
| **Pre-disclosure sessions** |  |  |
| - Session 1 |  | Added a statement in regard to CPS introduction; *“I work as a CPS at Jinja Regional Referral Hospital”* |
|  |  | - We reorganised the steps outlined in the example to start with “if the caregiver does not agree” followed by “if caregiver does agree.”  - Under “If caregiver does not agree” subsequent steps were reorganised for easy following and “*If other reasons, specify*” was added to explore other possible reasons for refusal to participate at that time. |
| - Step 1: Several changes were made to the questions and one was completely deleted. For example. | “Has (child’s name) ever had difficulties taking his/her HIV medicines as ordered? What  happened?” | “Has (child’s name) ever had difficulties taking his/her HIV medicines as ordered? *If yes,* What happened?” |
|  | “Do you have any health problems? Is anyone else in the family infected with HIV?” | “Are you affected by your child’s HIV status? Is anyone else in the family having a similar illness?” |
|  | “Who in the family knows about (child’s name) HIV status?” | “Who else knows about (child’s name) HIV status?” |
|  | “Has (child’s name) ever asked you about his/her illness?” | “Has (child’s name) ever asked you about his/her illness or reason for taking medicines?” |
|  | “Have you considered discussing (child’s name) HIV status with him/her?” | “Have you considered discussing (child’s name) HIV status with him/her? If no, why?” |
|  | “What would it take for you to feel more confident about taking your ARVs?” | Question was deleted. |
| - Follow-up Sessions | Frequency was not specified | Frequency specified as 2-weekly |
| **Disclosure sessions** |  |  |
| - Step 1. Assess the child’s readiness. |  | -Questions to assess Readiness of the Child or Adolescent (Glaser, 2016) were included. The CPS CPS will also complete the Pediatric Disclosure Readiness Assessment Checklist which was added as appendix 1. |
| - Step 2. One of the points to be covered during discussion was revised. |  | For adolescents or children at risk for unsafe sex, tell them about how to practice  Safe sex |
| **Post-disclosure sessions** |  |  |
| - Summary | Follow-up sessions with the family after 2 weeks, and then on a monthly basis for the first six months to assess impact of disclosure, to answer questions, and to help foster support between the child and the family. | Follow-up sessions with the family immediately (within 3 days) after disclosure, after 2 weeks, and then on a monthly basis, for the first six months to assess impact of disclosure, to answer questions, and to help foster support between the child and the family.  Note: This can be done through phone calls or face-to-face meetings as per the convenience of the clients and CPS. |
| - Step 2. Assess, review and answer child’s questions |  | “Assess child’s adherence to treatment” was added |
| - Step 3. Assess, review and answer caregiver’s questions. | Make referrals as necessary. | Make referrals as necessary to the social worker. |
